# Parenchymatous Gastric Haemorrhages

**Published:** 1913-05

**Authors:** C. A. Ewald

**Affiliations:** Berlin


### BUFFALO MEDICAL JOURNAL

VOLUME 68 MAY, 1913 No. 10

### ORIGINAL ARTICLES

### Parenchymatous Gastric Haemorrhages

BY DR. C. A. EWALD
Berlin

(Especially prepared and translated for this Journal)

THE usual causes of gastric haemorrhage, appearing as haematemesis with bloody stools or occult intestinal haemorrhage, are well known. The source of the haemorrhage in haematemesis is located either in the oesophagus or in the stomach itself. In the former location, the cause may be a peptic ulcer of the oesophagus, a fissure, an inflammatory process, a traumatism or the same lesions may involve a diverticulum. Still more frequent, however, are ruptured varices which occur under conditions of circulatory obstruction as in hepatic sclerosis or occlusion of the portal vein. J. Singer (*Med. Klin.* No. 22, 1912) reports a rare form of oesophageal haemorrhage with dysphagia and vomiting of clear blood and bloody mucus, in a case of chronic anaemia with contracted kidney. In such cases, the blood flowing first into the stomach, accumulates and is finally vomited, thus giving the appearance of primary gastric haemorrhage.

Just as blood from the oesophagus may accumulate in the stomach and appear as a gastric haemorrhage, so it may also enter the stomach from the intestine when the source is near the pylorus as in duodenal ulcer.

Haemorrhages from the stomach itself may be due to erosions of the mucous membrane, peptic ulcer, cancer, rarely aneurysm of the gastric arteries. Obviously, one must distinguish from naso-pharyngeal haemorrhages or from minor haemorrhages from a very irritable mucous membrane, as in chronic gastric catarrh, due to the introduction of the tube.

None of these haemorrhages will be considered here. Attention will be limited to those occurring without macroscopically or microscopically demonstrable lesions of the mucous membrane of either the stomach or oesophagus. Such haemorrhages vary greatly in amount and may be so severe as to be fatal.

One of the most frequent parenchymatous haemorrhages is vicarious menstruation. To the best of my knowledge, this was first observed by my former student, Prof. Kuttner (*Berliner Klin. Woch.*, Nos. 7 and 8, 1895). One occasionally finds blood in the stomach in women about to menstruate, usually in small quantity and observed only when the gastric contents are aspirated, but sometimes, genuine haematemesis occurs. Such haemorrhages either accompany frank menstruation or take its place. This peculiar occurrence was, at first doubted, but it has subsequently been confirmed by others. (Author, Congress for Internal Medicine, 1902, page 120.) The same kind of vicarious menstruation may occur from haemorrhoids (F. Boas, Diseases of the Stomach, 1911, page 365.)

Another source of parenchymatous gastric haemorrhage is chronic jaundice as in various severe lesions of the liver, portal obstruction, thrombosis and phlebitis. But, in these cases, haemorrhages beneath the skin and from other mucous membranes are usually coincident, so that we are not dealing with isolated gastric haemorrhage. We consider these conditions as due to abnormal penetrability of the vessel walls.

Severe haemorrhages of the stomach and intestines occur also in purulent appendicitis. Dieulafoy (*Sem. Med.*, 1901, page 53 and 67) first called attention to such haemorrhages, and others have subsequently reported similar cases (Author, *Deutsche Klin,* 1904, vol. 5, page 479; H. Riese, *Med. Klin.* 1910, No. 27), in which the closest examination of the gastric and intestinal mucous membrane showed no lesions. Although this is by no means established for all cases, we assume that we have to deal with a thrombosis of the mesenteric vain, and, especially, of those branches which course in the mesentery of the appendix.

Occasionally, the vomitus of tabetic cases is bloody. I do not allude to cases of so-called nervous vomiting of hysteria nor to cases of disease of the central nervous system which, toward the close of the vomiting, yield bloody masses. Under such circumstances, the source of the bleeding is clear: on account of the strain of the mucous membrane in gagging and vomiting, blood reaches the cavity of the stomach. I allude, rather, to the vomiting of black masses as occurs in the so-called crises noires. Vulpian (*Mal. des Syst. Nerveux,* Paris, 1879) and Charcot (*Gaz. Med.*, 1889, No. 39) have concluded the cause of these black crises is a trophic disturbance of the vessel walls analogous to what occurs in the subcutaneous haemorrhages of tabetics. Only in rare cases are frank alterations found in the form of ulcerative processes of the mucous membrane. Some time ago, I reported such a case in which a fresh peptic ulcer was manifest. But these are quite peculiar cases in the total of crises noires and this total amounts to not more than 2 or 3

per cent. of gastric crises of tabes. As a rule, the gastric mucous membrane is free from any lesion. A. Neumann (*Deutsche Zeit. für Nervenheilkunde,* vol. 29, page 398) explains these haemorrhages as due to rise of blood pressure in the course of the crisis, as has been generally held for the abdominal crises of tabes. But, in such cases, arterio-sclerotic changes are also present, arising even in fairly young individuals on a syphilitic basis, syphilis, indeed, being a frequent cause of tabes. G. Singer (1.c.) has recently reported several cases of quite sudden and severe gastric and intestinal haemorrhage in which no other cause than a luetic vascular lesion could be determined. However, these cases did not come to necropsy. The patients regarded the haemorrhages as purely nervous and of slight significance, although they vomited and passed by bowel considerable masses of blood. But, as Singer remarks, this is a manifestation that is characteristic of syphilis.

In the two following cases, observed by me, the existence of syphilis is, on the other hand, fully excluded by the history, the clinical manifestations and the post mortem findings so that there remains to be considered nothing but a parenchymatous haemorrhage. One case has already been reported in my Clinic of Gastric Diseases, third edition, page 417. The other is very recent. The first case was that of a man aged 24, admitted on account of moderate complaint of dyspepsia, with no previous gastric or syphilitic history. Quite suddenly, he had a severe gastric haemorrhage with bloody vomit and stools, repeated after a short intermission, so that the patient died. A careful necropsy revealed no lesion of either the gastric or the intestinal mucous membrane, nor arteriosclerosis nor any other organic change, indicative of syphilis.

The second case is especially noteworthy. A man aged 48, with no previous sickness, the father of healthy children, without alcoholic or syphilitic history, with negative Wassermann reaction; complained of moderate dyspeptic symptoms for the last three or four months but did not come to the clinic on their account, until he noticed a swelling in his abdomen and wished to know if it was serious. He proved to have an enormous spleen, extending two fingers' breadths to the right of the median line. It was smooth, not painful on pressure, the hilus could be plainly felt. No vascular murmurs could be heard over the tumor. There was no change in the liver, no icterus, no blood in the stomach contents or stools, no eggs of parasites, no changes in the ocular fundus; Wassermann reaction negative; large amount of indican in the urine but neither albumin, sugar nor urobilin. Passage of the stomach tube was strongly opposed but free HCl was absent in the stomach contents. The blood contained 110% of haemoglobin, 5,280,000 red cells, 20,000 white cells. The latter consisted of 84% multiforminuclears, 7% large lymphocytes, 2% small lymphocytes, 2% eosinophiles, 3% basophiles, 2% transitional forms. In short, there was no special alteration beyond a moderate polyglobulia.

By these fiindings, leucocythaemia, pseudo-leucocythaemia, hepatic sclerosis, Banti's disease, etc., were excluded and the diagnosis was limited to ecchinococcus cyst and thrombosis of the splenic vein. On the 14th day after admission, the patient, who had, up to this time, felt quite well, suddenly vomited 1240 c.c. of watery blood, without any premonitory symptoms. At the same time he passed several bloody stools. The splenic tumor noticeably diminished, thus dispelling the idea of an ecchinococcus cyst. After a few hours the patient died in collapse, in spite of every conceivable means of haemostasis and stimulation.

Section revealed a thrombosis of the splenic vein, evidently not recent. The thrombus had a central canal, as was easily seen in microscopic section. The venae gastricae breves and the gastro-epiploic vein were enormously distended. Occasionally parietal thrombi were found in the radicles of the portal vein, The liver, except for a slight fatty infiltration, was intact and, in particular (microscopically also), showed no interstitial inflammation. There was no ascites, peritonitis nor glandular swellings. The capsule of the spleen was smooth, the splenic pulp was rich in blood, tough, but not altered microscopically except that the vascular tissues were slightly thickened. In the whole course of the alimentary canal, there were no macroscopic nor miscroscopic lesions of the mucous membrane nor of the vessels, i.e., no erosions, ulcers, varicosities, etc.

Two questions suggest themselves: 1. What was the origin of the thrombosis of the splenic vein? 2. What was the direct cause of the haematemesis?

M. Simmonds (*Virchow's Arch.*, vol 207, page 360, 1912) has recently reported seven cases of isolated thrombosis of the splenic vein. In all, syphilis was demonstrated. Most had haemorrhages arising from ruptured varicosities or ulcers of the gastric mucosa. He concludes that the thrombosis developed from sclerotic changes in the vessels, occasioned by an unknown bacterial poison in connection with the syphilitic virus. But, in my case, syphilis was positively excluded. In a case of Edens (*Grenzgebiete der Med.* und. Chir., 1908, vol. 18, page 59) the splenic thrombosis was attributed to trauma, but nothing of the sort had occurred in the present case or, at least, the patient had given no history of an accident, although, with us, it is to the self interest of laborers to do so. The origin of the thrombosis, therefore, remains obscure.

2. The haematemesis cannot be explained except on the ground of a parenchymatous haemorrhage resulting from the markedly dilated gastric veins. The blood of the splenic vein whose normal course was obstructed, had found another path to the portal vein through the dilated venae gastricae breves, and the gastro-epiploic vein and through their radicles, the haemorrhages must have taken place. Whether the haemorrhage was by diapedesis or through minute ruptures of the vessels, remains in doubt; but, as we could find nothing in the way of a lesion, either macroscopically or microscopically, we are justified in considering the haemorrhage as parenchymatous. The severity of the haemorrhage and the absence of every apparent cause for its sudden occurrence are remarkable.

---

INTESTINAL HAEMORRHAGE SIMULATING ULCER. C. Capezzuoli, *Riv. Crit.* di Clin. Med., February, 1912. Male, aged 30, good family and previous history, except for two previous haemorrhages, two weeks and one week before first examination, judging from symptoms. Red cells 880,000, blood in faeces for ten days, otherwise clinical pathology negative. After nineteen days, slight haematemesis, indications of considerable bleeding, death. All sources of haemorrhage excluded but diapedesis. Pathologic changes as described by Dr. Ewald.

---

FIVE-INCH HAIR PIN IN CAECUM, L. McGavin, *Lancet,* Jan. 11, 1913, reports a case in a physician who used the straightened pin to make an application to his own throat and accidentally swallowed it. No symptoms attended its passage through the upper alimentary canal. The radiogram showed the pin clearly but gave no idea as to its location. The pin was removed by section.

---

ABDOMINAL PREGNANCY WITH LIVING CHILD. J. S. Horsley of Richmond reported the case to the Southern Surg. and Gyn. Assn. (*Critic and Guide,* March, 1913). The patient was colored, previously normal, and the condition was recognized by the emptiness of the uterus, at about the completion on pregnancy. A male child, weighing six pounds was removed by section. 103 cases of abdominal pregnancy were found in the literature from 1809 to 1912, in five of which mother and child were alive and well one year after operation.

